# Spontaneous transverse colon volvulus

**DOI:** 10.11604/pamj.2013.14.160.2073

**Published:** 2013-04-25

**Authors:** Landolsi Sana, Gassara Ali, Helmi Kallel, Baklouti Amine, Saadaoui Ahmed, Elouer Mohamed Ali, Chaeib Wajdi, Mannaï Saber

**Affiliations:** 1 Department of general surgery, Jendouba's Hospital, Tunisia

**Keywords:** Volvulus, Transverse colon, spontaneous

## Abstract

We report a case of spontaneous transverse colon volvulus in a young healthy woman. It constitutes an unusual case since it occurred in a young healthy woman with a subacute onset and no aetiological factor has been found. Its diagnosis is still challenging. Prompt recognition with emergency intervention constitutes the key to successful outcome.

## Introduction

The transverse colon volvulus is an uncommon cause of bowel obstruction. It constitutes a surgical emergency since it can lead to bowel infarction, peritonitis, and death if not diagnosed at once. We report a case of a spontaneous transverse colon volvulus in a young healthy woman.

## Patient and observation

A 39-year old woman presented with a three-day history of constipation and progressive abdominal pain without nausea or vomiting. The last bowel movement had been three days ago. There was no significant past medical history especially of chronic constipation, psychiatric disease, neurologic disease, or abdominal surgery. Physical examination revealed a moderate distension of the abdomen without signs of peritonitis. The abdomen was tympanic to percussion. There were no umbilical or groin hernias. Digital rectal examination demonstrated an empty rectal vault without intraluminal masses.

**Figure 1:**
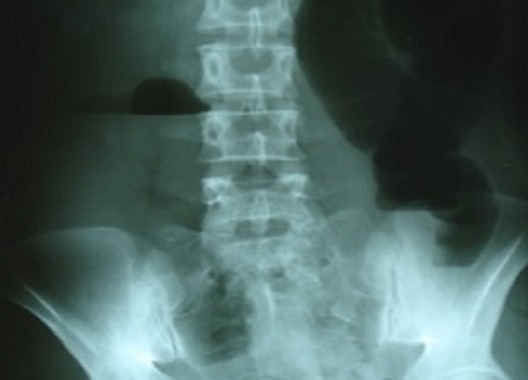
Abdominal X-rays showing a dilated transverse colon with a “U-shaped” loop in the left upper abdomen.

**Figure 2:**
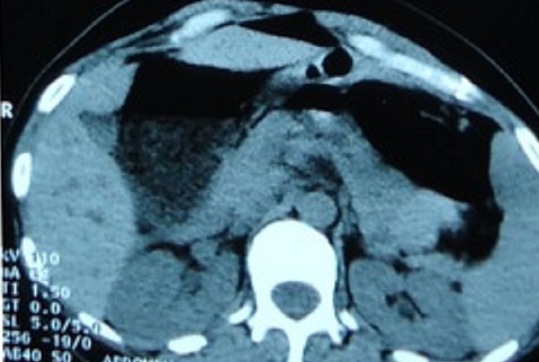
Computed tomography revealing dilatation of the right colon and the transverse colon with a cut-off near the splenic flexure.

Blood investigations showed normal full blood count, urea, electrolytes, and clotting profile. The abdominal X-ray revealed a large bowel obstruction with a “U-shaped” loop in the left upper abdomen (Figure 1). The computed tomography demonstrated a dilatation of the right colon and the transverse colon with a cut-off near the splenic flexure (Figure 2). No signs of malignancy were found.

**Figure 3:**
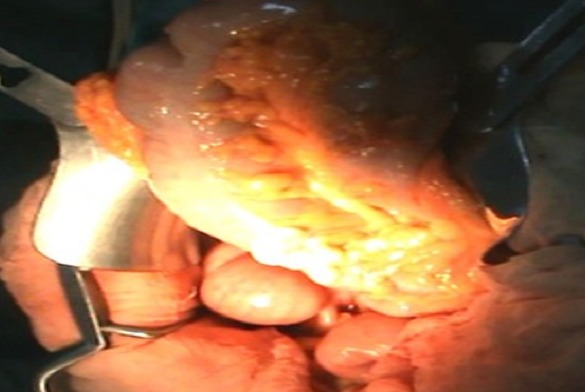
Intra operative view demonstrating transverse colon volvulus. The point of twist was located in the left upper quadrant of the abdomen.

An emergency laparotomy was performed. Intra operative findings (Figure 3) were of a transverse colon volvulus rotated in a three hundred and sixty degrees clockwise direction on its mesentery. The point of twist was found in the left upper quadrant. The bowel was intact without signs of ischemia. A significant disparity in the size of the obstructed proximal and collapsed distal colon to the site of the volvulus was noticed. The transverse colon was mobile and increased in length. The volvulus was delivered into the incision and detorsed. An extended right hemicolectomy was carried on with end-to-side ileo-colic anastomosis.

The postoperative course was uneventful. Discharge from hospital was five days following admission. Histologically, the appearances were consistent with a sub acute progressive transverse colon volvulus. No acute inflammation, infarction, granulomas, dysplasia, malignancy, or vascular abnormality was noticed.

## Discussion

We reported a case of spontaneous transverse colon volvulus in a healthy young woman. She had no past medical history of chronic constipation, psychiatric disease, neurologic disease, or abdominal surgery. Intra operatively, the transverse colon was mobile with an increased length. There was no sign of malignancy.

Only 3 to 5% of all cases of intestinal obstruction are caused by colonic volvulus [1]. Among them, transverse colon is involved in 2 to 4% [2, 3] versus 43 to 80% and 15 to 43% respectively for the sigmoid colon and the coecum [3]. A male predominance is noticed [3]. Its pathogenesis is not completely understood yet. Predisposing factors are congenital, physiological, and mechanical [1, 4]. The two congenital properties thought to cause a volvulus are redundancy and non-fixation [5] as found in our case. Chagas disease can result in a megacolon increasing colonic length. Physiological causes include high-roughage diet and large bowel distension secondary to chronic constipation. This constipation is associated with psychiatric or neurologic diseases [3]. Mechanical causes include previous volvulus of the transverse or the sigmoid colon, distal colonic obstruction, adhesions, malposition of the colon following previous surgery, mobility of the right colon, inflammatory strictures, and carcinoma [1, 4]. Other factors have been reported such as Chilaiditis syndrome [4], Clostridium difficile pseudomembranous colitis [6], and impaired intestinal motility associated with pregnancy.

Our patient presented with subacute transverse volvulus. This progressive onset of the symptoms can delay the diagnosis and the treatment thus resulting in progressing to the acute fulminating type with bowel infraction, peritonitis, and even death [4]. The subacute onset is characterized by massive abdominal distension in the setting of mild abdominal pain without rebound tenderness, nausea, or vomiting [1]. The leukocyte count is normal secondary to the lack of ischemia at early stages. Unlike our case, this type usually occurs in elderly patients affected by several comorbidities and bed-bound. Patients with the acute fulminating type of presentation have a sudden onset of severe abdominal pain, rebound tenderness, vomiting, little distension, and rapid clinical deterioration. Bowel sounds are initially hyperactive becoming absent later on [1, 7].

In our case, the distribution of the large bowel dilatation in abdominal X-ray could have raised the possibility of volvulus since it revealed a “U-shaped” loop with the apex pointing under the left hemidiaphragm. The diagnosis of transverse colon volvulus is difficult because it does not have the same classically recognizable radiographic features as sigmoid and coecal volvulus. It is usually made intra operatively. In the subacute type, the achievement of an early diagnosis through computed tomography is advised [7].

Whereas sigmoid volvulus can be decompressed by colonoscopy, transverse colon volvulus must be surgically detorsed in emergency [4]. Resection constitutes the treatment of choice to prevent recurrence [1, 4]. In fact, detorsion alone or associated with colopexy has a higher rate of recurrence than resection [1, 4]. The incidence of recurrent volvulus after previous resection and primary anastomosis varies between 22% and 36% [3]. Therefore, some authors recommend considering a subtotal colectomy in the presence of a megacolon, instead of partial resection of the involved bowel segment [3]. This resection is carried on with or without primary anastomosis dependent on the aspect of the colon, the existence or not of peritonitis, and the state of health of the patient. Our patient had an extended right hemicolectomy with a primary anastomosis. The post operative course was uneventful.

## Conclusion

Transverse colon volvulus is rare. Its Diagnosis is challenging. Prompt recognition with emergency intervention constitutes the key to successful outcome.
